# Game vs. Practice Differences in External Load in U16 and U18 Women’s Basketball Players

**DOI:** 10.3390/sports13090296

**Published:** 2025-09-01

**Authors:** Damjana V. Cabarkapa, Dimitrije Cabarkapa, Dora Nagy, Laszlo Balogh, Tamas Laczko, Laszlo Ratgeber

**Affiliations:** 1 D2 Lab, 21000 Novi Sad, Serbia; 2 Faculty of Physical Education and Management in Sport, Singidunum University, 11010 Belgrade, Serbia; 3 Jayhawk Athletic Performance Laboratory, Wu Tsai Human Performance Alliance, Department of Health Sport and Exercise Sciences, University of Kansas, Lawrence, KS 66045, USA; 4 Faculty of Health Sciences, Doctoral School of Health Sciences, University of Pecs, 7602 Pecs, Hungary; 5 Faculty of Health Sciences, Institute of Physiotherapy and Sport Science, University of Pecs, 7602 Pecs, Hungary; 6 Center for Basketball Methodology and Education, 7621 Pecs, Hungary; 7 Institute of Sport Science, University of Debrecen, 4032 Debrecen, Hungary; 8 Department of Sport Games, Hungarian University of Sports Science, 1123 Budapest, Hungary

**Keywords:** distance, player load, acceleration, deceleration, training stress, speed, jump

## Abstract

The purpose of the present study was twofold: (i) to examine within-group differences in external load metrics during practice and official competition, and (ii) to examine between-group differences in external load metrics across the U16 and U18 levels of play. A total of thirty-six female athletes participated in the present study, of which nineteen were U16 and seventeen were U18 basketball players. The athletes wore an inertial measurement unit system (Kinexon) sampling at 20 Hz during practice and official games. The average values for each external load metric across ten practices and five games were used for performance analysis. Dependent and independent *t*-tests were used to examine within- and between-group statistically significant differences, respectively (*p* < 0.05). The findings reveal that the external load placed on the athletes during the game (e.g., distance covered, average speed, total number of accelerations and decelerations) was considerably greater than the external load during practice sessions, both on the U16 and U18 levels of play. Conversely, while the game-induced external load remained consistent across the two competitive levels, U18 players tended to spend more time and cover more distance in low-speed zones than in high-speed zones during practice, compared to their U16 counterparts, suggesting their superior movement efficiency.

## 1. Introduction

Basketball is a dynamic team sport characterized by repetitive high-intensity movements (e.g., accelerations, decelerations, and change of direction), separated by short periods of low-intensity activities (e.g., walking, jogging) [1,2,3,4]. As basketball players advance to higher competitive levels, the physical, technical, and tactical demands of the game progressively increase. Thus, in order to effectively prepare youth athletes for these escalating on-court competitive demands, it is crucial to understand the training loads that they encounter during practice sessions and games, which may allow coaches and sports scientists to design impactful training regimens that directly align with the challenges of high-level competition.

Training load has been previously defined as “any physiological work performed by an athlete during both training sessions or competition and its subsequent impact on the athlete” [5,6,7,8]. Depending on whether the training load is viewed from the perspective of the task itself or the individual’s physiological response to it, it can be classified as either external or internal [6,9,10]. Specifically, external load measures the absolute physical demands placed on the athlete (e.g., total distance, number of accelerations and decelerations), while internal load reflects the athlete’s physiological response to this specific stimulus (e.g., heart rate, rating of perceived exertion) [2,6,10,11].

In recent years, advances in technology have made external load monitoring increasingly popular in an applied sports setting, providing precise data on the physical demands placed on athletes during training and competition [10,12]. Specifically, in basketball, inertial measurement units (IMU) or local positioning systems (LPS) are being widely used to quantify external load and provide practitioners with a plethora of metrics such as maximum or average speed and number of accelerations, decelerations, and jumps performed by each athlete individually, as well as overall as a team [12]. By tracking these performance-related parameters over time, sports practitioners, in conjunction with the coaching staff, can adequately adjust the recovery protocols and periodization strategies to optimize athletes’ readiness, decrease the likelihood of overuse injuries, and reduce the levels of fatigue.

To date, multiple research reports have highlighted the use of IMU and LPS technology for external load monitoring in the game of basketball [13,14,15,16]. For example, Montgomery et al. [14] examined the physical and physiological responses of junior male basketball players during practice drills and games using the IMU system (i.e., Catapult) and heart rate monitoring device. The researchers have found that official gameplay yielded substantially greater physical load (~85%), average heart rate (~12%), and oxygen demands (~30%) than 5-on-5 scrimmage [14]. However, no notable differences in the physical and physiological responses were observed between defensive and offensive drills during practice sessions [14]. On the other hand, Brandao et al. [13] have found contradictory findings, showing that male basketball players competing at the U17 level of play experience similar training loads during practice sessions and official games. Yet, it should be noted that external load tends to be highly dependent on the type of activity being performed (e.g., sprints, jumps, high-intensity specific movements), as well as whether the activity involves the ball or not [15]. In addition, the external load experienced by professional male basketball players tends to differ significantly across various competitive levels. Specifically, Ujakovic et al. [16] indicated that higher-ranked teams (i.e., ACB League) are exposed to considerably greater external loads (i.e., player load) compared to those competing at the lower level of play (i.e., ABA League) (i.e., 10.70 vs. 8.44 AU). While these findings offer valuable insights, there is still a lack of research pertaining to the external load experienced by youth basketball players, especially among female athletes, which remains an understudied population in the scientific literature.

Therefore, to bridge an existing gap in the scientific literature, the aim of this study was two-fold: (i) to examine within-group differences in external loads metrics (e.g., total acceleration and decelerations, high speed distance, average and maximal speed, jumps per minute, total distance covered) during practice and official competition in U16 and U18 female basketball players, and (ii) to examine between-group differences in external load metrics across these two levels of play (U16 vs. U18). Based on previously published research, it is hypothesized that notable differences will be observed between practice and game competitive scenarios, as well as between the two levels of play.

## 2. Materials and Methods

### 2.1. Participants

A total of thirty-six female athletes volunteered to participate in the present study, from which nineteen were U16 (age = 14.8 ± 0.6 years; height = 174.1 ± 7.8 cm; body mass = 61.7 ± 8.5 kg) and seventeen U18 (age = 16.8 ± 1.1 years; height = 178.8 ± 6.0 cm; body mass = 69.9 ± 9.4 kg) basketball players. Both groups of athletes competed on the top-tier national level of play in each respective category. All athletes were (i) current members of their respective teams, (ii) had more than three years of basketball playing experience, (iii) were cleared to participate in team training sessions and games by their respective sports medicine staff, (iv) had no musculoskeletal injuries that would limit or impair their basketball performance, and (v) participated in structured team strength and conditioning and basketball-specific training sessions more than four times per week. The data used in the present study were collected as part of regular institutional procedures for which individual and/or parental consents were obtained. The approval to use the data for research purposes was received from both the basketball academy and the University’s Institutional Review Board.

### 2.2. Procedures

The data collection procedures were based on previously published research reports [17,18,19]. Each athlete wore an IMU (Kinexon Prevision Technologies, Version 1.0, Munich, Germany) during consecutive practice sessions and games over a three-week time span. This technology has been demonstrated as a valid testing modality for tracking walking, jogging, sprinting, jumping, as well as change of direction movements in team sports such as basketball [20]. The sampling frequency of the device was 20 Hz. Based on manufacturer recommendations, the IMU was positioned between the scapulae in a tightly fitted west or as a part of a sports bra to minimize movement artifact. The location of the device remained consistent throughout the data collection process. The live recordings for each practice and game (i.e., regular season competitive period) started simultaneously for all athletes and ended at the same time, including warm-up protocols and brief water breaks or timeouts. The recordings began 2–3 min before the practice session or an official game and concluded immediately afterward. The reported external loads during the game excluded a 15-min half-time break. The selection of external load metrics examined in this investigation was based on previously published research reports [21,22,23] and it included: (i) total and average distance per minute covered in high-speed (HS), moderate-speed (MS), and low-speed (LS) zones; (ii) maximal and average speed; (iii) total and average active time spent in HS, MS, and LS zones; (iii) total and average number of accelerations, decelerations, jumps, and change of directions (COD); and (iv) accumulated acceleration load (ACC; i.e., sum of all accelerations performed in x, y, and z-axis). The data was collected over ten practice sessions in which each athlete participated non-stop from start to end of the training session (i.e., 87.9 ± 4.7 min), and five official games in which each athlete was part of a starting lineup or was constantly included in playing rotation (i.e., >20 min/game of playing time). The average values for each external load metric across ten practice sessions and five official games were used for performance analysis purposes.

### 2.3. Statistical Analysis

Shapiro-Wilk’s test and Q-Q plots were used to corroborate that the assumption of normality was not violated. Descriptive statistics (i.e., mean and standard deviation) were calculated for each dependent variable of interest. Dependent *t*-tests were used to examine differences between practice and game external load metrics, and independent *t*-tests were used to conduct between-group comparisons (U16 vs. U18). Due to the within-group sample size (n < 20), Hedge’s *g* was used to calculate the measure of effect size to avoid small-sample bias (i.e., *g* = 0.2 is a small effect, *g* = 0.5 is a moderate effect, and *g* > 0.8 is a large effect) [24]. All statistical analysis procedures were completed in SPSS (Version 28.0.1.1; Chicago, IL, USA). The α level of *p* < 0.05 was used as a criterion for statistical significance.

## 3. Results

At the U16 level of basketball competition, significantly greater total distance (*p* < 0.001; *g* = 1.573), distance per minute (*p* < 0.001; *g* = 5.265), HS distance (*p* < 0.001; *g* = 1.867), MS distance (*p* < 0.001; *g* = 2.110), LS distance (*p* < 0.001; *g* = 0.679), HS distance per minute (*p* < 0.001; *g* = 3.146), MS distance per minute (*p* < 0.001; *g* = 4.741), LS distance per minute (*p* < 0.001; *g* = 2.493), maximal speed (*p* < 0.001; *g* = 1.354), average speed (*p* < 0.001; *g* = 5.000), HS time (*p* < 0.001; *g* = 2.003), MS time (*p* < 0.001; *g* = 1.944), LS time (*p* < 0.012; *g* = 0.408), total acceleration (*p* < 0.001; *g* = 1.431), accelerations per minute (*p* < 0.001; *g* = 3.959), total decelerations (*p* < 0.001; *g* = 1.625), decelerations per minute (*p* < 0.001; *g* = 3.881), and ACC (*p* < 0.013; *g* = 1.247) was found during games when compared to practice sessions. On the other hand, no significant differences in external load metrics between practice and games at the U16 level of play were found in total active time (*p* = 0.091), total COD movements (*p* = 0.501), COD per minute (*p* = 0.081), total jumps (*p* = 0.371), and jumps per minute (*p* = 0.582).

While LS distance (*p* = 0.155) and maximal speed (*p* = 0.768) did not reach the level of statistical significance, a comparison of external load metrics at the U18 level of play revealed that total distance covered (*p* < 0.001; *g* = 1.403), distance per minute covered (*p* < 0.001; *g* = 4.899), HS distance (*p* < 0.001; *g* = 2.314), MS distance (*p* < 0.001; *g* = 5.108), HS distance per minute (*p* < 0.001; *g* = 3.693), MS distance per minute (*p* < 0.001; *g* = 5.343), LS distance per minute (*p* < 0.001; *g* = 1.163), average speed (*p* < 0.001; *g* = 4.314), HS time (*p* < 0.001; *g* = 2.344), MS time (*p* < 0.001; *g* = 1.878), total accelerations (*p* < 0.001; *g* = 1.233), acceleration per minute (*p* < 0.001; *g* = 4.525), total COD movements (*p* < 0.006; *g* = 0.660), COD per minute (*p* < 0.001; *g* = 1.000), total decelerations (*p* < 0.001; *g* = 1.280), decelerations per minute (*p* < 0.001; *g* = 4.525), and AAC (*p* = 0.013; *g* = 1.019) were considerable greater during the game than practice. Conversely, total active time (*p* < 0.001; *g* = 1.274), LS time (*p* = 0.007; *g* = 0.787), total jumps (*p* < 0.001; *g* = 1.756), and jumps per minute (*p* < 0.001; *g* = 1.264) were significantly lower during the game when compared to team practice at the U18 level of play.

When comparing external load metrics during practice between U16 and U18 competitive levels, U18 athletes demonstrated notably greater total distance covered (*p* < 0.040; *g* = 0.713), LS distance (*p* < 0.001; *g* = 1.378), LS distance per minute (*p* < 0.001; *g* = 1.560), total active time (*p* = 0.045; *g* = 0.695), MS time (*p* = 0.027; *g* = 0.735), LS time (*p* = 0.004; *g* = 1.022), total jumps (*p* < 0.001; *g* = 2.059), and jumps per minute (*p* < 0.001; *g* = 1.931) when compared to their U16 counterparts. Moreover, U18 basketball players during practice had lower HS distance (*p* < 0.001; *g* = 1.508), HS distance per minute (*p* < 0.001; *g* = 1.624), maximal speed (*p* < 0.001; *g* = 1.140), HS time (*p* < 0.001; *g* = 1.380), total COD (*p* = 0.049; *g* = 0.667), and COD per minute (*p* = 0.037; *g* = 1.000) than U16 basketball players. However, no statistically significant differences in practice external load metrics between U16 and U18 competitive levels were found in distance per minute covered (*p* = 0.850), MS distance (*p* = 0.109), MS distance per minute (*p* = 0.916), average speed (*p* = 0.855), total accelerations (*p* = 0.491), acceleration per minute (*p* = 0.118), total decelerations (*p* = 0.654), decelerations per minute (*p* = 0.139), and AAC (*p* = 0.797).

Lastly, no statistically significant differences in any external load metrics during the game were found between U16 and U18 levels of play: total distance covered (*p* = 0.667), distance covered per min (*p* = 0.558), HS distance (*p* = 0.473), MS distance (*p* = 0.786), LS distance (*p* = 0.782), HS distance per minute (*p* = 0.682), MS distance per minute (*p* = 0.495), LS distance per minute (*p* = 0.416), maximal speed (*p* = 0.225), average speed (*p* = 0.548), total active time (*p* = 0.602), HS time (*p* = 0.385), MS time (*p* = 0.898), LS time (*p* = 0.570), total accelerations (*p* = 0.501), acceleration per minute (*p* = 0.730), total COD (*p* = 0.889), COD per minute (*p* = 0.745), total decelerations (*p* = 0.745), decelerations per minute (*p* = 0.274), total jumps (*p* = 0.548), jumps per minute (*p* = 0.367), and AAC (*p* = 0.297). Descriptive statistics (i.e., mean and standard deviation) for each dependent variable of interest are presented in Table 1.

## 4. Discussion

To the best of our knowledge, this is the first study focused on examining within-group and between-group differences in external load metrics during practice and competition among U16 and U18 female basketball players. Overall, the findings reveal that the external load placed on athletes during the game (e.g., total distance covered, average speed, total number of accelerations and decelerations) was considerably greater than the external load during practice, both on the U16 and U18 levels of play. On the other hand, when examining between-group differences (U16 vs. U18), the results reveal that U18 players during practice covered more distance, had greater active time and jump count, and showed larger values for LS metrics than U16 players. However, they also attained lower maximal speed, total and average COD movements, and HS metrics than their U16 counterparts. In addition, unlike the aforementioned findings, no significant differences between the two levels of play were observed in any external load variables during the live competitive scenarios, with all metrics being similar in magnitude.

Previous literature has found similar findings, indicating that external load tends to be significantly greater during live competition when compared to regular practice sessions [14,25,26,27,28]. Specifically, research across various team sports, such as soccer, football, and ice hockey [27,28,29,30], as well as basketball [14,25], has emphasized the increased physical and physiological demands during a game, which are further intensified by the psychological stress that athletes experience during competition (e.g., decision making, crowd). For example, Brown et al. [25] found that high-minute players on a female collegiate basketball team exhibited significantly greater external loads during competition than during practice (e.g., cumulative player load, number of accelerations, decelerations, and COD), which seems to be in direct agreement with the findings of the present investigation. Similar observations were made by Montgomery et al. [14], who found that junior male basketball players accumulated considerably greater physical load during live basketball games when compared to 5-on-5 practice scrimmage (i.e., 279 ± 58 vs. 171 ± 85 AU), which further supports our results obtained on a cohort of U16 and U18 female basketball players. These variations in external load demands during training and live competitive scenarios may be largely attributed to the intentional periodization strategies employed by coaches, strength and conditioning practitioners, and sports scientists during the in-season competitive period, designed to minimize cumulative fatigue and ensure adequate recovery for athletes. In addition, it should be noted that a similar trend has been detected in the scientific literature when examining the internal load variables [11,31]. For example, Moreira et al. [31] showed that official matches elicited significantly greater cortisol levels and ratings of perceived exertion than the simulated games in professional male basketball players, further supporting the differences in the overall stress placed on the athletes between practice and live game competitive scenarios.

Besides within-group comparisons of the external load placed on the athletes during practice and competition, it is also important to acknowledge the progress that naturally occurs through different stages of athletic development. Specifically, as athletes get older and start participating at higher levels of play, significant increases in the training load demands are likely to occur [32,33]. The findings of the present study indicate that U18 female basketball players are exposed to significantly greater external load during practice than their U16 counterparts (e.g., total distance covered, MS and LS time, total jump count, total active time), which aligns with the aforementioned notion pertaining to the athletes’ developmental progress. This can be largely attributed to the differences in the tactical-technical strategies implemented by U16 and U18 coaches. For example, older athletes may engage in more complex and physically demanding practice drills or training scenarios to better develop tactical decisions, skill proficiency, and physical capacity needed for future competitive demands. In line with this observation, Wrigley et al. [33] found that U18 male soccer players experienced significantly higher weekly training loads during the in-season competitive period when compared to their U16 and U14 counterparts. Similarly, Rauter et al. [32] found a considerable increase in both training volume (58.7%) and training load (49.0%) among male youth cyclists from ages 15 to 18, further confirming the results obtained in the present study. However, unlike the previously mentioned research reports [32,33], the present investigation found that external loads during the game tend to remain relatively consistent between U16 and U18 levels of play, with almost all external load metrics being similar in magnitude (e.g., maximal speed, total number of accelerations, decelerations, and jumps). Besides being attributed to the sport-specific demands (e.g., basketball vs. soccer) and the athlete’s sex (i.e., female vs. male), this discrepancy in the findings may be attributed to the fundamental nature of the competition. Precisely, game regulations (e.g., duration, court dimensions, number of opponents) remain consistent across age groups, while practice sessions are flexible and typically vary in order to meet the individual developmental needs of athletes. Thus, it is likely that the consistent nature of these competitive elements contributes to the stability of these external load metrics during games.

Another interesting finding observed in the present investigation pertains to the difference in HS and LS time and distance, as well as the number of COD movements performed during practice between the U16 and U18 levels of play. Our results indicate that U18 players tend to spend more time and cover more distance in LS than in HS zones than their U16 counterparts, suggesting their superior movement efficiency. While further research is warranted on this topic, this assumption aligns with the previously published research reports [34,35], indicating that athletes competing at higher levels of play tend to have superior knowledge of the game, allowing them to be more efficient in performing high-intensity actions and ultimately reducing the overall physical and physiological demands experienced during practice or competition. Moreover, a lower number of total COD and COD per minute observed in the present study within the U18 cohort of athletes further confirms the aforementioned assumption pertaining to movement efficiency. Thus, due to being older and having greater tactical-technical knowledge of the game, the U18 athletes may know when high-intensity efforts or COD maneuvers are needed to beat the defender down the court or to position themselves in the optimal offensive or defensive position.

While offering a deeper insight into the external load experienced by female youth basketball players during both training and competitive scenarios, the present study is not without limitations. The internal load, such as the rating of perceived exertion or heart rate, corresponding to the external load measures obtained, was not incorporated into the analysis procedures. Hence, future research should focus on combining the measures of internal and external load in order to obtain a more comprehensive understanding of the physiological demands that female youth athletes experience during training and competition. So, future research should examine how these differences vary across different positions (e.g., guard, center), as well as whether the training load of the youth athletes remains similar between starting players and their substitutes, alongside expanding the participant sample size.

## 5. Conclusions

The findings of the present study reveal that the external load placed on amateur female basketball players during the game (e.g., total distance covered, average speed, total number of accelerations and decelerations) was considerably greater than the external load during practice sessions, both on the U16 and U18 levels of play. On the other hand, while game-induced external load demands were found to remain relatively consistent across the two competitive levels, U18 players tend to spend more time and cover more distance in LS than in HS zones during practice than their U16 counterparts, suggesting their superior movement efficiency. Also, these findings may suggest that external loads during practice should be adjusted to reflect the external loads experienced during competition in order to optimize performance and minimize the risk of injury occurrence due to insufficient readiness. However, the adjustments should be individualized and context-specific, ensuring a balance between athlete recovery and long-term development (e.g., periodization). Moreover, these findings highlight the importance of continuous training load monitoring in all levels of women’s basketball play, which allows coaches, strength and conditioning coaches, and sports scientists to make well-informed decisions regarding the training regimens and in-season periodization strategies directed toward optimizing athletes’ on-court performance.

## Figures and Tables

**Table 1:** Descriptive statistics, mean and standard deviation ($\overset{\text{-}}{x}$ ± SD), and statistical comparisons (i.e., practice vs. game; U16 vs. U18) for each external load metric examined in the present study.

Variable [unit]	Practice (U16)	Game (U16)	Practice (U18)	Game (U18)
Total distance covered [m]	3439.8 ± 258.0	4416.9 ± 839.2 *	3599.8 ± 179.2 ^#^	4305.1 ± 688.1 *
Distance covered per min [m/min]	52.0 ± 2.8	68.4 ± 3.4 *	51.8 ± 2.9	69.2 ± 4.1 *
HS distance [m]	727.2 ± 72.6	1039.3 ± 224.9 *	608.9 ± 84.5 ^#^	985.9 ± 214.3 *
MS distance [m]	1203.9 ± 104.5	1723.9 ± 332.4 *	1255.9 ± 82.0	1695.4 ± 89.9 *
LS distance [m]	1417.8 ± 195.3	1595.9 ± 315.4 *	1647.6 ± 127.1 ^#^	1569.7 ± 236.7
HS distance per minute [m/min]	11.1 ± 1.4	16.0 ± 1.7 *	8.9 ± 1.3 ^#^	15.8 ± 2.3 *
MS distance per minute [m/min]	18.1 ± 2.0	26.9 ± 1.7 *	18.2 ± 1.6	27.3 ± 1.8 *
LS distance per minute [m/min]	21.3 ± 1.5	24.8 ± 1.3 *	23.5 ± 1.3 ^#^	25.2 ± 1.6 *
Maximal speed [km/h]	22.0 ± 0.6	20.8 ± 1.1 *	21.2 ± 0.8 ^#^	21.2 ± 0.9
Average speed [km/h]	3.1 ± 0.2	4.1 ± 0.2 *	3.1 ± 0.2	4.2 ± 0.3 *
Total active time [s]	4004.2 ± 388.8	3832.0 ± 646.1	4228.6 ± 226.6 ^#^	3729.5 ± 505.3 *
HS time [s]	210.4 ± 21.6	306.9 ± 64.6 *	177.8 ± 25.7 ^#^	288.3 ± 61.5 *
MS time [s]	617.4 ± 59.3	868.8 ± 173.0 *	654.6 ± 38.5 ^#^	861.8 ± 151.2 *
LS time [s]	1757.5 ± 298.4	1897.3 ± 382.3 *	2018.4 ± 196.0 ^#^	1833.5 ± 266.8 *
Total accelerations [#]	318.4 ± 26.0	402.1 ± 78.5 *	324.2 ± 23.6	385.5 ± 66.2 *
Acceleration per minute [#]	4.8 ± 0.3	6.2 ± 0.4 *	4.6 ± 0.3	6.2 ± 0.4 *
Total COD [#]	23.4 ± 5.2	24.5 ± 6.8	20.2 ± 4.3 ^#^	24.2 ± 7.4 *
COD/minute [#]	0.4 ± 0.1	0.4 ± 0.2	0.3 ± 0.1 ^#^	0.4 ± 0.1 *
Total decelerations [#]	304.1 ± 25.5	399.5 ± 79.0 *	307.8 ± 22.4	372.1 ± 67.4 *
Decelerations/minute [#]	4.6 ± 0.3	6.2 ± 0.5 *	4.4 ± 0.3	6.0 ± 0.4 *
Total jumps [#]	43.3 ± 7.4	41.8 ± 12.0	66.1 ± 14.1 ^#^	44.1 ± 10.6 *
Jumps/minute [#]	0.6 ± 0.1	0.66 ± 0.1	0.9 ± 0.2 ^#^	0.7 ± 0.1 *
AAC [AU]	341.9 ± 36.3	426.8 ± 89.2 *	344.7 ± 28.6	398.4 ± 68.8 *

Note: AAC—accumulated acceleration load; COD—change of direction; HS—high speed; MS—moderate speed; LS—low speed; AU—arbitrary unit; (*) significantly different when compared to practice; (#) significantly different when compared to U16 practice (*p* < 0.05).

## Data Availability

The data presented in this study are available on request from the corresponding author.
